# Role of Natural Compounds and Target Enzymes in the Treatment of Alzheimer’s Disease

**DOI:** 10.3390/molecules27134175

**Published:** 2022-06-29

**Authors:** Shanshan Wang, Xianbo Kong, Zhangjing Chen, Guopin Wang, Juan Zhang, Jing Wang

**Affiliations:** 1 Department of Convalescent Area, Ningbo Psychiatric Hospital, Ningbo 315040, China; wangshanshan0024@outlook.com (S.W.); kongxianbo3542@outlook.com (X.K.); 2 Department of Cadre Ward, 906 Hospital of the Chinese People’s Liberation Army, Ningbo 315040, China; chenzhangjing5520@outlook.com; 3 Department of Five Disease Areas, Ningbo Psychiatric Hospital, Ningbo 315040, China; wangguopin0024@outlook.com; 4 Department of Inspection, Ningbo Psychiatric Hospital, Ningbo 315040, China; zhangjuan0025@outlook.com; 5 Department of Neurology, Sanya Central Hospital, Hainan Third People’s Hospital, Sanya 572000, China

**Keywords:** Alzheimer’s disease, natural compound, mechanism of enzyme, management, inhibition activity

## Abstract

Alzheimer’s disease (AD) is a progressive neurological condition. The rising prevalence of AD necessitates the rapid development of efficient therapy options. Despite substantial study, only a few medications are capable of delaying the disease. Several substances with pharmacological activity, derived from plants, have been shown to have positive benefits for the treatment of AD by targeting various enzymes, such as acetylcholinesterase (AChE), butyrylcholinesterase (BuChE), β-secretase, γ-secretase, and monoamine oxidases (MAOs), which are discussed as potential targets. Medicinal plants have already contributed a number of lead molecules to medicine development, with many of them currently undergoing clinical trials. A variety of medicinal plants have been shown to diminish the degenerative symptoms associated with AD, either in their raw form or as isolated compounds. The aim of this review was to provide a brief summary of AD and its current therapies, followed by a discussion of the natural compounds examined as therapeutic agents and the processes underlying the positive effects, particularly the management of AD.

## 1. Introduction

Alzheimer’s disease (AD) is a severe, chronic, and progressive neurological illness that causes memory and cognitive loss and eventually death [1]. Dementia has become a major public health problem in both developed and developing countries as a result of the aging population and its fast-rising incidence [2]. Aging, cholinergic pathways, environmental factors, head injury, genetic factors, mitochondrial dysfunction, and immune system dysfunction are some common causes of the development of AD [3]. The most prevalent form of dementia is AD, which is a progressive neurological condition [4]. The reported deaths from AD increased by more than 145% [5]. According to the most recent estimates, dementia prevalence will double in Europe by 2050 and triple globally. AD is pathologically defined by the presence of amyloid plaques, hyperphosphorylated tau proteins, and neurofibrillary tangles; however, oxidative–nitrative stress, endoplasmic reticulum stress, mitochondrial dysfunction, inflammatory cytokines, pro-apoptotic proteins, and altered neurotransmitter levels are all common etiological attributes in its pathogenesis. Rivastigmine, memantine, galantamine, and donepezil are Food and Drug Administration-approved medications for the treatment of symptoms associated with AD [6]. The cellular phase of AD occurs concurrently with the accumulation of amyloid, causing tau pathology to spread. Heritable variables account for 60–80% of the risk of AD [7]. A decrease in brain acetylcholine (ACh) levels is implicated in the pathophysiology of cognitive dysfunction occurring in AD. The inhibition of ACh catabolic enzymes, such as acetylcholinesterase (AChE) and butyrylcholinesterase (BuChE), can contribute to an increase in ACh levels. It has been hypothesized that the inhibition of AChE and BuChE may contribute to countering the formation of Aβ plaques and, therefore, represents a disease-modifying strategy principle, but no conclusive evidence was obtained to confirm this hypothesis [8].

AChE inhibition is one of the most used treatment approaches; however, it only provides symptomatic alleviation and has a mild disease-modifying impact. Antioxidant and vitamin treatment, stem cell therapy, hormone therapy, selective phosphodiesterase (PDE) inhibitors, inhibition of β-secretase, γ-secretase and Aβ aggregation, suppression of tau hyperphosphorylation, and intracellular neurofibrillary tangles are examples of non-cholinergic therapeutic methods. In a number of preclinical and clinical investigations, medicinal plants have been found to have anti-AD efficacy [9,10]. Ethnobotany plays a significant role in the identification of anti-AD compounds from botanicals in China and the far east, but maybe less so in Europe. Traditional Chinese medicine has been employed in the treatment of AD in China. A variety of medicinal plants have been shown to diminish the degenerative characteristics associated with AD, either in their crude form or as isolated substances [1]. The consumption of bioactive compound-rich foods or the administration of bioactive compound extracts can have a preventive impact against a variety of pathophysiological diseases. Various sources of bioactive chemicals are employed in the treatment of AD. We have just covered the most frequent options.

It is reported that dietary supplements might help to heal the disorders. Nutraceuticals are food-based extracts of chemicals that offer health advantages. Nutraceuticals are ingested in concentrated forms such as tablets, capsules, and drinks, and they have no negative effects, even at large doses. To avoid the negative side effects of the currently available medications, researchers are concentrating their efforts on identifying natural bioactive chemicals found in foods that can be used to treat AD [11,12,13,14]. The consumption of bioactive compound-rich foods or the administration of bioactive compound extracts can have a preventive impact against a variety of pathophysiological diseases. Although there are other sources of bioactive chemicals used in the treatment of AD, we only included the most widely available. The impact of numerous bioactive chemicals found in widely consumed foods on AD has been reviewed and addressed in this section. The aim of this review is to evaluate the role of natural compounds and the mechanism of enzymes for the management of AD. An extensive literature review (by inclusion of natural compounds and target enzymes, and the exclusion of synthetic compounds) was carried out, and published articles from PubMed, Scifinder, Google Scholar, Clinical Trials.org, and the Alzheimer Association reports were thoroughly examined in order to combine information on the various ways to battle AD. Therefore, in this article, we focus on reviewing the potential target and small natural compounds targeting various molecular mechanisms for the management of AD.

## 2. Natural Compounds and Alzheimer’s Disease

Natural products and their molecular frameworks have a long history of serving as important starting points for medicinal chemistry and drug development [15]. Recent studies have discussed the many therapeutic properties of natural products, such as their ability to improve sleep [16], hypolipidemic activity and anticancer effects [17,18], protective effects against viral pneumonia and anti-inflammatory effects [19], anticancer and antioxidative effects [20,21], neuroprotective effects [22], antioxidative stress and anti-asthmatic effects [23,24,25], alleviating the effect of skin inflammation [26], and anti-Trypanosoma effects [27]. However, natural products can cause pulmonary and central nervous system (CNS) irritation [28], developmental toxicity [29,30], nephrotoxicity and hepatotoxicity [31], and allergic responses [32,33]. There are presently no effective drugs available to treat ND. In traditional medicine, ashwagandha is used to treat general debility, nervous weariness, insomnia, and memory loss [34]. In studies, these natural compounds have been shown to exhibit biological qualities, such as antioxidant, anti-inflammatory, and antiapoptotic effects. In vitro and in vivo studies have confirmed the use of natural products in a variety of preclinical models of ND. Phytoconstituents, such as polyphenolic antioxidants found in herbs, fruits, nuts, and vegetables, as well as marine and freshwater flora, are examples of natural products. These phytoconstituents have the ability to prevent several NDs, such as AD [35,36]. Consumption of these substances at adequate quantities may have promising benefits in the prevention of AD [37].

## 3. Inhibition of Acetylcholinesterase Activity Using Natural Compounds

**Figure 1:**
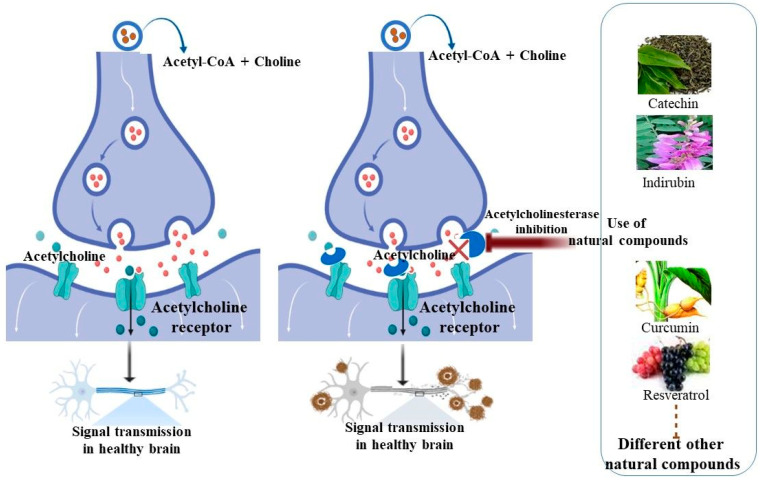
The inhibition process of AChE using natural compounds. AChE inhibitors such as natural compounds bind to the AChE enzyme and prevent the breaking down of ACh, causing ACh to accumulate in nerve synapses and impair neurotransmission.

Acetylcholinesterase (AChE) is a serine hydrolase that hydrolyzes the neurotransmitter acetylcholine (ACh) into acetic acid and choline. The ellipsoidal structure of AChE has three binding sites: catalytic anionic (Ser200, Glu334, and His440), the aromatic gorge, and the peripheral anionic site (Tyr70, Asp72, Tyr121, Trp279, and Tyr334), where inhibitory chemicals engage. AChE inhibitors (AChEI) bind to this enzyme and prevent it from breaking down ACh, causing ACh to accumulate in nerve synapses and impair neurotransmission. Many medicinal compounds targeting AChE have been developed based on this mechanism of action [38,39,40,41]. As a result, using AChEI to treat symptoms associated with cholinergic imbalances in AD seemed a sensible strategy. AChE and the cholinergic system, on the other hand, appear to have broader impacts in AD. Many useful compounds that demonstrate a wide spectrum of pharmacological action against cholinesterase enzymes have been discovered through phytochemical research of various therapeutic plants [42]. Dihydroberberine and macelignan potently and effectively inhibited AChE with IC50 values of 1.18 and 4.16 µM, respectively [43]. *Quercus suber* cork and corkback ethanol–water extracts have been proven to be remarkable antioxidants with interesting AChE inhibitory activity [44]. Using the in vitro Ellman’s technique, extracts, fractions, and compounds from *Calceolaria talcana* and *Calceolaria integrifolia* showed substantial inhibitory effects on AChE activity. The most active samples were derived from the ethyl acetate extract, which inhibited AChE in a mixed-type manner (69.8 and 79.5% at 100 and 200 μg/mL, respectively) [45]. It was also reported that between 0 and 5 min, AChE inhibition increased as the time spent exposed to Malathion increased [46]. The edible component of the *Garcinia parvifolia* fruit has the potential to be a natural source of antioxidants and anti-AD agents [47]. Phytochemicals continue to enter clinical trials or give leads for the development of new therapeutic medicines [48]. The use of natural products or nutraceutical chemicals has emerged as a potential preventative therapy approach, as most medications focusing on specific targets have failed to establish a medical cure. Nutraceutical substances have the benefit of a multitarget strategy, tagging several biochemical locations in the human brain, as compared to the single-target action of most AD medications [49]. In the last decade, more than 200 potential therapeutic candidates have failed during clinical trials, indicating that the illness and its causes are likely to be complicated. Medicinal herbs and herbal therapies are gaining popularity as complementary and alternative interventions to create medication candidates for AD. Several scientific investigations have documented the use of numerous medicinal plants and their main phytochemicals in the treatment of AD [50,51]. The increasing collection of epidemiological and experimental research shows that eating fruits and vegetables protects the brain from the negative consequences of oxidative stress, neuroinflammation, and aging. These benefits are mediated by antioxidant, anti-inflammatory, and other beneficial phytochemical components present in plants [52]. However, it was also reported that consistent use of coffee, tea, and dark chocolate (cacao) may boost brain health and lower the incidence of age-related neurodegenerative disease (ND). Caffeine’s mode of action is based on the antagonism of several adenosine receptor subtypes. Theobromine and theophylline, which are downstream xanthine metabolites, may also contribute to the therapeutic benefits of coffee, tea, and cocoa on brain function [53]. Tea is said to have powerful antioxidant effects. Flavonoids, tannins, caffeine, polyphenols, boheic acid, theophylline, theobromine, anthocyanins, gallic acid, and ultimately epigallocatechin-3-gallate, which is regarded the most potent active element, are all abundant. Tea catechins, which are flavonoid phytochemicals that target common risk factors, including obesity, hyperlipidemia, hypertension, cardiovascular disease, and stroke, may help to reduce the risk of AD [54]. The effects and probable mechanisms of numerous widely eaten phytochemicals on neuropathology and AD outcomes are discussed in this study. We propose that frequently eating bioactive phytochemicals from a range of fruits and vegetables reduces age and insult-related neuropathology in AD, based on available data. This holistic approach to nutraceuticals paves the way for future research and clinical trials, which are expected to provide outcomes based on medical evidence. The molecular mechanism of AChE was described in Figure 1, along with the inhibition process of AChE using natural compounds.

## 4. Inhibition of BACE1 Activity Using Natural Compounds

In 1991, the amyloid hypothesis was proposed. It claimed that extracellular amyloid deposits are the primary cause of AD [55]. β-secretase (BACE1) was found to be responsible for the creation of β-amyloid (Aβ) observed in AD [56]. Aβ is a type I transmembrane protein with a large extracellular domain and a short cytoplasmic portion that is generated from an amyloid precursor protein (APP). As a result of alternative splicing, several distinct APP isoforms exist, ranging in length from 695 to 770 amino acid residues [57]. Neurons create a considerable quantity of APP. However, it is normally digested quite fast. APP may be cleaved by six distinct enzymes, namely, α-, β-, δ-, η- and θ-secretase and meprin β [58]. In AD, APP is cleaved alternatively in endosomal compartments by the successive action of the integral membrane β- and γ-secretase, releasing Aβ from the APP [59,60]. β-secretase divides APP, producing a 100 kDa soluble N-terminal APP ectodomain (APPs) and a 12 kDa membrane-tethered C-terminal fragment with 99 or 89 amino acid residues, depending on whether it cleaves at Asp1 or Glu11 of the APP. Under healthy settings, BACE1 mostly cleaves APP at the Glu11 location, resulting in the non-amyloidogenic form C89 and truncated Aβ production [61]. Verubecestat, lanabecestat, atabecestat, umibecestat, and elenbecestat are in II/III phase clinical trials as BACE1 inhibitors [61]. The IC50 values for these drugs were found to be 2.2 nM for verubecestat [62], 0.6 nM for lanabecestat [63], and 1.0–2.6 nM for atabecestat [64]. The reduction of Aβ in CSF depended on the daily dose and it was shown that verubecestat reduces Aβ in CSF by 50–75% at a 12 mg dose and 80–90% at a 40 mg dose [65]. Lanabecestat reduces 63% at a 15 mg dose and 79% at a 50 mg dose [66]; atabecestat reduces 50% at a 5 mg dose and 80–85% at a 30 mg dose [67]; and umibecestat reduces 95% at a 15 mg dose [68].

**Figure 2:**
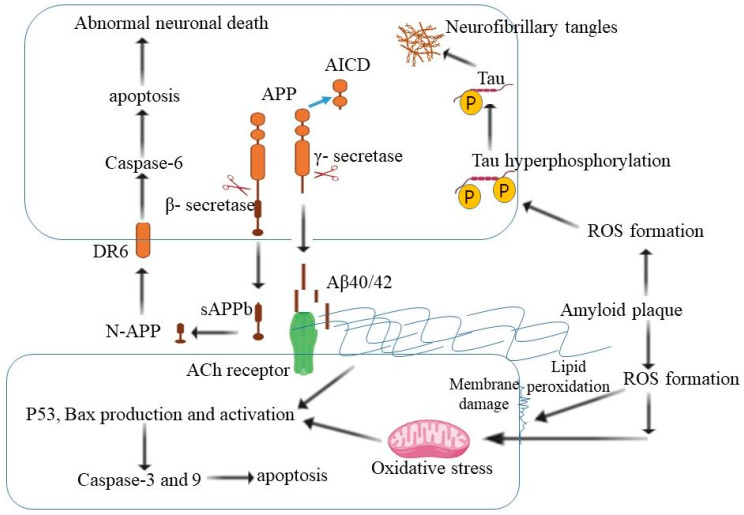
The molecular mechanism of β- and γ-secretase for the processing of APP. The extracellular amyloid deposits are the primary cause of AD. The natural compounds may be alternative agents that have β- and γ-secretase inhibition for the management of AD (APP—amyloid precursor protein, AICD—APP intracellular domain, Aβ—β-amyloid, ROS—reactive oxygen species).

Natural products, particularly those used in traditional Chinese medicine, offer a safety advantage, since they have been used in humans for a long period [69]. Inhibiting BACE1 has been intensively researched as a possible AD disease-modifying medication. Clinical failures with BACE inhibitors have risen steadily. As a result, researchers are thinking about natural compounds as potent drug therapies for the management of AD-targeting BACE1. The natural compounds catechins may also aid people with AD by decreasing the formation of amyloid plaques and enhancing their cognitive ability [54]. To explore natural BACE1 inhibitors, isoflavones, including genistein, formononetin, glycitein, daidzein, and puerarin, were studied and found to be potent for AD management [70]. Compounds such as 2,2′,4′-trihydroxychalcone acid, quercetin, and myricetin have been demonstrated to efficiently inhibit BACE1 activity at lower dosages [71]. The compounds deoxyneocryptotanshinone, salvianolic acid A and salvianolic acid C were found to have good inhibition potential against BACE1, with IC50 values of 11.53 ± 1.13, 13.01 ± 0.32 and 9.18 ± 0.03 μM, respectively [72]. The natural compounds may be alternative agents that have β- and γ-secretase inhibition for the management of AD in the future. The molecular mechanism of β- and γ-secretase is described in Figure 2.

## 5. Inhibition of Monoamine Oxidase Activity Using Natural Compounds

Monoamine oxidases (MAOs) are flavoproteins that catalyze the oxidative deamination of biogenic and xenobiotic amines in the outer mitochondrial membrane. There are two isoforms of MAO in mammals (MAO-A and MAO-B), which may be identified by their substrate selectivity and susceptibility to certain inhibitors. Although both isoforms are found in most tissues, their presence in the CNS and their capacity to metabolize monoaminergic neurotransmitters have shifted the focus of MAO research to the adult brain’s functions. MAO activity has been linked to neurological and mental illnesses, as well as NDs [73]. Some inhibitors of the enzyme have showed promise in the treatment of a variety of NDs, such as Parkinson’s disease and AD. MAO inhibitors may be effective in regulating the outcome of stroke and other tissue damage linked with oxidative stress, since the process catalyzed by MAO creates hydrogen peroxide, which is a source of hydroxyl radicals [73,74,75]. MAO inhibitors might be used to treat AD [76]. While MAO-A inhibitors (e.g., chlorgyline, moclobemide, and lazabemide) are efficient antidepressants and anxiolytic medications, MAO-B inhibitors (e.g., l-deprenyl, pargyline, and rasagiline) are used to treat NDs such as Parkinson’s and AD. Natural products have become appealing targets for researchers, owing to the need for novel MAO inhibitors due to the negative effects of existing drugs. Many investigations have shown that flavonoid, xanthone, alkaloid, and coumarin derivatives from herbal sources have high MAO inhibitory action, making them ideal models for synthetic MAO inhibitors [77]. Curcumin and ellagic acid suppressed MAO activity; however, greater half-maximum inhibitory doses of curcumin (500.46 nM) and ellagic acid (412.24 nM) were needed when compared to the known MAO-B inhibitor selegiline. It has been discovered that curcumin and ellagic acid suppress MAO activity in both competitive and noncompetitive ways. These natural chemicals have the potential to be a source of MAO inhibitors, which are utilized in the treatment of Parkinson’s disease and other NDs [78]. Chelerythrine was reported to have an IC50 of 0.55 µM for inhibiting an isoform of recombinant human MAO-A. Chelerythrine was a reversible competitive MAO-A inhibitor (Ki = 0.22 µM) with a substantially higher potency than the marketed medication toloxatone, with an IC50 value of 1.10 µM [79]. The natural O-methylated flavonoid, with strong potency (IC50 33 nM; Ki 37.9 nM) and >292-fold selectivity against human MAO-A (vs. MAO-B), is a novel therapeutic lead for the treatment of NDs [80]. The other natural compounds, such as morin (IC50 = 16.2 µM), alizarin (IC50 = 8.16 µM), and fisetin (IC50 = 7.33 µM), were notable MAO inhibitors with MAO-A selectivity [80]. As compared to known drugs, natural products have fewer side effects and are efficient for the inhibition of these enzymes. Researchers are looking for natural products that have very good potential to inhibit these enzymes, which may be helpful for future treatment options.

Finally, there are certain known natural compounds listed in Table 1. These compounds were found to be suitable for the inhibition of targeted enzymes during in silico, in vitro and in vivo studies.

## 6. Conclusions

The reviewed compounds have the ability to lessen the symptoms of AD. With the increasing average life expectancy, it is critical to find and create novel molecules easily capable of preventing AD. Several natural compounds and phytochemicals have shown promise in clinical research for AD management. Several medications appear to be useful for AD treatment in clinical studies. Natural substances in the early stages of study require more investigation to determine their medicinal potential for AD management. It is critical to recognize that alternative therapies for AD may be widely supported in medical research.

## Figures and Tables

**Table 1:** List of several natural compounds that have potential to inhibit AChE, BuChE, BACE1 and MAOs activity during in silico, in vitro, and in vivo studies.

S.No.	Compound	Pub Chem ID	Properties	Work Type	Therapeutic Actions/Function	Reference
1.	Apigenin	5280443	Antioxidant and antiinflammatory	in vitro	Decrease Aβ burden	[1,81]
				in vivo (mouse model)	induced neurogenesis	
2.	Dibenzo[1,4,5]thiadiazepine	71358659	antioxidant	in vitro (neuroblastoma cells)	neuroprotective and antioxidant properties	[82]
3.	Berberine	2353	anti-inflammatory	in vitro (rat model)	inhibition of AChE	[83,84]
4.	Catechin	9064	antioxidant	in vivo (rat model of AD)	inhibition of AChE	[85]
5.	Genistein	5280961	Antioxidant and anti-inflammatory	in silico and in vitro (model of AD)	inhibition of human monoamine oxidase A and B	[86,87]
6.	Hesperidin	10621	antioxidant and anti-inflammatory	in silico and in vivo (rat model of AD)	inhibition of BACE1 and Aβ aggregation	[88,89,90]
7.	Morin	5281670	antioxidant, anti-inflammatory and neuroprotective	(MC65 cells)	BACE1, γ-secretase, Aβ fibrillogenesis, amyloid plaque, and tau hyperphosphorylation	[91,92]
8.	Naringenin	932	anti-inflammatory	in vitro (rat model)	decrease inflammatory cytokines	[93]
9.	Withanone	21679027	neuroprotective	in vivo (rat model of AD)	decrease Aβ fibril formation	[1,94]
10.	Dehydroevodiamine	9817839	anti-inflammatory	rat brain slices against AD	inhibition of tau phosphorylation	[95]
11.	Huperzine A	449069	neuroprotective	Alzheimer transgenic mouse model	reduces the level of Aβ	[96]
12.	N-methylasimilobine	197017	Antioxidant	in vitro	inhibition of AChE	[97]
13.	Isorhynchophylline	3037048	neuroprotective	rat model	restore Aβ–induced cognitive impairment	[98]
14.	Palmatine	19009	anti-inflammatory and anti-neurodegenerative	in vitro, in vivo	inhibit tau aggregation	[99]
15.	Sanguinarine	5154	Antitumor properties	in vitro	inhibition of AChE	[100]
16.	Taspine	215159	anti-inflammatory	in vitro	inhibition of AChE	[101]
17.	Indirubin	10177	antioxidant and anti-inflammatory	in silico	inhibition of AChE	[102,103]
18.	Rutaecarpine	65752	anti-inflammatory	in silico	inhibition of Caspase 8	[104]
19.	Ajmalicine	441975	antihypertensive	in silico	inhibition of BACE1	[105]
20.	Resveratrol	445154	Antioxidant	in vitro and in vivo (AD models)	neuroprotective role in AD	[106]
21.	Curcumin	969516	antioxidant, anticarcinogenic, anti-inflammatory, antiangiogenic	in vivo and in vitro	inhibition of AChE	[107]
22.	Resveratrol	445154	Antioxidant	in vitro	inhibition of MAOA for AD treatment	[108]
23.	Genistein	5280961	Antioxidant and anti-inflammatory	in vitro	anti-AD activities	[70]
24.	Quercetin	5280343	Antioxidant	-	Anti-BACE1 Activity	[71]
25.	Ellagic acid	5281855	antioxidant, antimutagenic, and anticancer properties	in vitro	MAO inhibitor for ND treatment	[78]
26.	Chelerythrine	2703	anti-inflammatory	in vitro	MAO-A inhibitor	[79]

## Abbreviations

AD	Alzheimer’s disease
AChE	Acetylcholinesterase
BuChE	Butyrylcholinesterase
Ach	Acetylcholine
CNS	Central nervous system
ND	Neurodegenerative disease
Aβ	β-amyloid
APP	Amyloid precursor protein
MAOs	Monoamine oxidases
